# Performance Assessment of the Network Reconstruction Approaches on Various Interactomes

**DOI:** 10.3389/fmolb.2021.666705

**Published:** 2021-10-05

**Authors:** M. Kaan Arici, Nurcan Tuncbag

**Affiliations:** ^1^ Graduate School of Informatics, Middle East Technical University, Ankara, Turkey; ^2^ Foot and Mouth Diseases Institute, Ministry of Agriculture and Forestry, Ankara, Turkey; ^3^ Chemical and Biological Engineering, College of Engineering, Koc University, Istanbul, Turkey; ^4^ School of Medicine, Koc University, Istanbul, Turkey

**Keywords:** protein-protein interactions, interactome, network reconstruction, heat diffusion, personalized PageRank, prize-collecting Steiner forest, pathway reconstruction

## Abstract

Beyond the list of molecules, there is a necessity to collectively consider multiple sets of omic data and to reconstruct the connections between the molecules. Especially, pathway reconstruction is crucial to understanding disease biology because abnormal cellular signaling may be pathological. The main challenge is how to integrate the data together in an accurate way. In this study, we aim to comparatively analyze the performance of a set of network reconstruction algorithms on multiple reference interactomes. We first explored several human protein interactomes, including PathwayCommons, OmniPath, HIPPIE, iRefWeb, STRING, and ConsensusPathDB. The comparison is based on the coverage of each interactome in terms of cancer driver proteins, structural information of protein interactions, and the bias toward well-studied proteins. We next used these interactomes to evaluate the performance of network reconstruction algorithms including all-pair shortest path, heat diffusion with flux, personalized PageRank with flux, and prize-collecting Steiner forest (PCSF) approaches. Each approach has its own merits and weaknesses. Among them, PCSF had the most balanced performance in terms of precision and recall scores when 28 pathways from NetPath were reconstructed using the listed algorithms. Additionally, the reference interactome affects the performance of the network reconstruction approaches. The coverage and disease- or tissue-specificity of each interactome may vary, which may result in differences in the reconstructed networks.

## Introduction

Computational approaches improve our understanding about the mechanisms of perturbations, effects of drugs, and functions of genes in the biological system by interpreting multiple “omic” data and reducing their complexity (Liu et al., 2020; Paananen and Fortino, 2020). Integrative network analysis approaches are used to interpret the complex interactions between “omic” entities as a whole beyond the list of molecules. The impact of an alteration in any omic entity, for example, upregulated or downregulated genes or mutated or phosphorylated proteins, may not be local; rather, it diffuses to the distant sites of the interactome.

Many pathway databases cataloged the molecular interactions. Each database explains interactions *via* different approaches. KEGG (Kanehisa et al., 2017) provides annotated pathways, while Reactome (Jassal et al., 2020) gives detailed information on components and the reactions. Additionally, integrated interactomes such as HIPPIE, ConsensusPathDB, and STRING combine multiple resources to come up with a weighted interactome. There are several scoring schemas to measure the reliability of interactions such as MI-score and IntScore. These methods combine different weights including the number of publications, detection method, or network topology (Turinsky et al., 2011; Kamburov et al., 2012; Kamburov et al., 2013; Alanis-Lobato et al., 2017; Szklarczyk et al., 2019). Although the combination of multiple resources improves the quality of the interactomes, it still does not completely solve the bias toward well-studied proteins or the artifacts from high-throughput experiments (Žitnik et al., 2013; Caraus et al., 2015; Skinniderid et al., 2018; Vitali et al., 2018). Besides the false positives, interactomes are not complete and have false negatives which are the undetected interactions. To complete the missing parts in the interactome and to detect spurious interactions, several prediction approaches have been employed using network topology (Alkan and Erten, 2017), link prediction, protein structures (Singh et al., 2006; Tuncbag et al., 2012; Mosca et al., 2014; Segura et al., 2015; Yerneni et al., 2018; Ietswaart et al., 2021), or additional data such as gene expression (Cannistraci et al., 2013; Lei and Ruan, 2013; Hulovatyy et al., 2014; Szklarczyk et al., 2021). For example, Interactome3D uses the structural knowledge in PDB and homology-based prediction to construct a highly accurate interactome (Mosca et al., 2013). The main limitation of proteome-wide structural interactome construction is the number of structurally resolved protein complexes.

**FIGURE 1:**
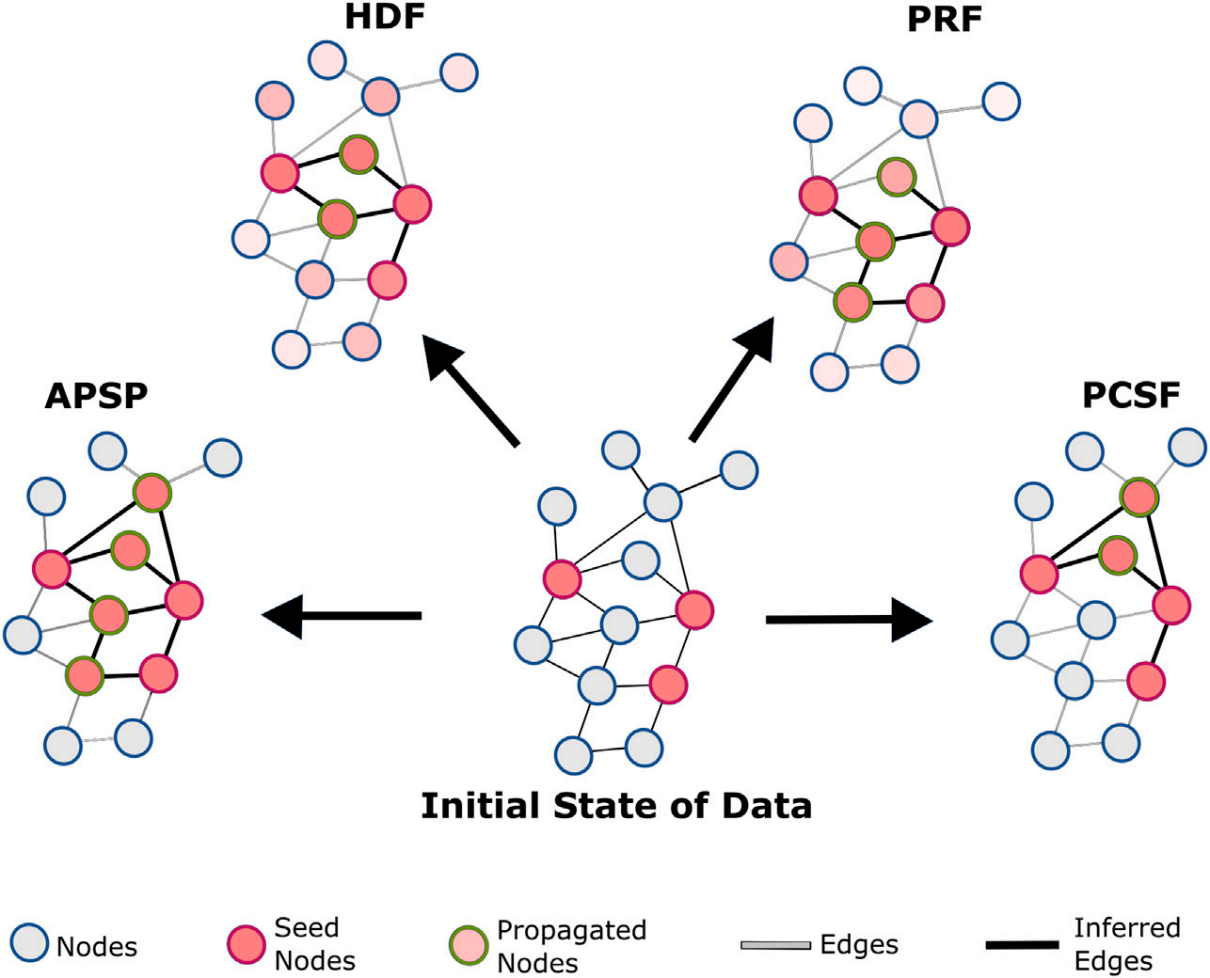
Conceptual representation of reconstruction algorithms: all-pair shortest paths (APSP), personalized PageRank with flux (PRF), heat diffusion with flux (HDF), and prize-collecting Steiner forest (PCSF). In the APSP, the subnetwork is reconstructed with the union of all shortest paths between seed nodes. HDF diffuses the heat that initially belongs to seed nodes. After limited steps of transfer, the heat of the nodes is used for flux score calculations for edges. PRF uses a personalized PageRank algorithm to find the probability of nodes after randomly walking in the reference interactome and calculates flux scores. PCSF finds the optimum forest to link seed nodes either directly or through intermediate nodes. The union of optimum forests reconstructs subnetworks.

Network reconstruction approaches aim to transform the list of seed genes/proteins into their interactome-wide impact based on the topological proximity. Steiner trees/forests, statistical models, and network propagation with random walk or heat diffusion systems have been frequently used in omics data integration with the molecular interactions (Leiserson et al., 2015; Cowen et al., 2017; SeahSen et al., 2017) or identifying disease-associated pathways, subnetworks, or modules (Paull et al., 2013; Kim et al., 2015; Silverbush et al., 2019). These approaches construct context-specific subnetworks under a certain condition such as disease association or for revealing the impact of an external stimulus such as drug treatment or pathogen infection (Braunstein et al., 2019; Tabei et al., 2019). Recently, DriveWays (Baali et al., 2020), MEXCOwalk (Ahmed et al., 2020), iCell (Malod-Dognin et al., 2019), ModulOmics (Silverbush et al., 2019), and Omics Integrator (Tuncbag et al., 2016b) predicted the cancer driver modules. MEXCOwalk implements a random walk on the reference interactome by using mutation frequencies and their mutual exclusivity for the identification of the cancer driver modules. ModulOmics uses protein–protein, regulatory, and gene co-expression networks together with mutual exclusivity of mutations to identify highly functional driver modules. Omics Integrator solves the prize-collecting Steiner forest problem to construct optimal subnetworks from the single- or multi-omic datasets. Omics Integrator was applied to several conditions from cancer driver network construction (Dincer et al., 2019) and to viral infection modules in the host organisms (Sychev et al., 2017). iCell uses the matrix factorization to integrate multi-omics datasets with tissue-specific interactomes. In this study, we compared the performance of four network reconstruction approaches, all-pairs shortest path (APSP), personalized PageRank with flux (PRF), heat diffusion with flux (HDF), and prize-collecting Steiner forest (PCSF), on six different interactomes. A conceptual representation of these methods is illustrated in Figure 1. We did not consider the methods in this comparison that modify the underlying interactome or reconstruct regulatory networks using gene expression, such as ARACNe (Lachmann et al., 2016), GENIE (Fontaine et al., 2011), and INFERELATOR (Madar et al., 2009). APSP merges the shortest paths between pairs of nodes in the seed list. HDF implements the heat diffusion process by transferring the initial heat of the seed list to their neighbors. PRF applies a random walk to find the nodes most relevant to the seed list. We calculate the edge flux in both HDF and PRF based on the resulting node weights. PCSF finds an optimal forest that connects the seeds either directly or by adding intermediate nodes. We evaluated the performance of these algorithms on a gold standard dataset containing 32 curated pathways in the NetPath database using different metrics such as precision, recall, and MCC values. The performance of each network reconstruction approach is highly dependent on the reference interactomes. Additionally, each method has its own strengths and limitations. We found that the interactomes have some critical differences that can significantly affect the performance of network reconstruction approaches, such as their edge weight distributions, the bias toward some well-studied proteins, their coverage of disease-associated proteins, and the structurally resolved interactions. APSP has the highest recall and the lowest precision, while PRF, HDF, and PCSF have more balanced and comparable performance in precision and recall. Among them, PCSF performed the best in terms of the F1 score, which represents the balance between the precision and the recall. Overall, our study presents an extensive comparison of the selected network reconstruction approaches and shows the impact of the input interactome in their performance. This comparison presenting the strong and weak aspects of the interactomes and reconstruction approaches has the potential to be beneficial to the field.

## Methods

### Reference Interactomes

We used PathwayCommons v12 (Rodchenkov et al., 2019), iRefWeb v13 (Turinsky et al., 2011), HIPPIE v2.2 (Alanis-Lobato et al., 2017), ConsensusPathDB (Kamburov et al., 2013), STRING (Szklarczyk et al., 2019, 2021), and OmniPath (Ceccarelli et al., 2020) for the interactome comparison and the assessment of subnetwork inference approaches. We mapped the names of proteins in interactomes (nodes) to their reviewed Uniprot identifiers (The UniProt Consortium, 2019). The statistics of the interactomes are listed in Table 1. Some interactomes have confidence scores, which represent how real an interaction is. PathwayCommons and OmniPath do not have confidence scores. iRefWeb uses the MI-scoring scheme, which considers multiple parameters including experimental detection methods. HIPPIE v2.2 and ConsensusPathDB (Release 34) have confidence scores on edges calculated based on their own scheme (Kamburov et al., 2012; Alanis-Lobato et al., 2017). We filtered the STRING interactome by recalculating confidence scores considering only the experiment and database scores (von Mering et al., 2005).

**TABLE 1:** Reference interactomes and their statistics.

Interactome	Number of proteins	Number of interactions	Confidence score
iRefWeb v13.0	11,295	80,351	Yes
PathwayCommons v12	18,536	1,126,072	No
HIPPIE v2.2	15,984	369,584	Yes
ConsensusPathDB	17,269	359,201	Yes
STRING v11	8,992	229,306	Yes
OmniPath	6,549	35,684	No

### Interactome Comparison Metrics

We compared the reference interactomes at both the node and edge levels using different metrics, namely, the overlap coefficient, correlation of edge confidence scores, inclusion of disease-associated proteins, and overlap with the pathway edges. The overlap coefficient is a similarity measure for two given datasets, ***S***_***1***_ and ***S***_***2***_, which can be node sets or edge sets of graphs or information coming from a database. The overlap coefficient was calculated using Eq. 1 for pairwise comparison of interactomes and coverage of varied knowledge (Simpson, 1966; Kuzmin et al., 2016) as follows:

$$\mathrm{overlap}\left(S_{1},S_{2}\right)=\frac{\left|S_{1}\cap S_{2}\right|}{\min\left(\left|S_{1}\right|,\left|S_{2}\right|\right)}$$ (1)

We compared each pair of interactomes, ***G(V***_***G***_ ***, E***_***G***_ ***, c(e***_***G***_ ***))*** and ***H(V***_***H***_ ***, E***_***H***_ ***, c(e***_***H***_ ***))***, where ***V*** is the node set and ***E*** is the edge set, and ***0≤c(e)≤1***, where ***c(e)*** is the confidence score of an edge. The node-level similarity of the given interactomes were calculated using the overlap coefficient by applying Eq.1 where ***V***_***G***_ is used as ***S***_***1***_ and ***V***_***H***_ is ***S***_***2***_. Likewise, the edge level overlap coefficient in each pair of interactomes is determined using Eq. 1 where ***E***_***G***_ and ***E***_***H***_ are assigned as ***S***_***1***_ and ***S***_***2***_, respectively.

Next, we explored the structurally known protein-protein interactions in each reference interactome using the overlap coefficient. Interactome INSIDER has 4,150 interactions from PDB and 2,901 interactions from Interactome3D (Meyer et al., 2018). The edge level overlap coefficient between each reference interactome (***G***) and each structural interactome (***H***) is calculated using Eq. 1.

The interactomes and network reconstruction methods are frequently used for revealing cancer driver modules. We downloaded the 568 cancer driver genes (CDGs) from intOGen (Martínez-Jiménez et al., 2020). The overlap coefficient between CDGs (***S***_***1***_) and proteins in each reference interactome (***S***_***2***_) is calculated using Eq. 1. Additionally, the number of publications about each CDG and the degree centrality of the CDGs are analyzed to find out the bias of the interactomes toward well-studied or cancer-associated proteins.

The overlap of each reference interactome (**G**) with the known interactions in 171 pathways in KEGG (**H**) is calculated using Eq. 1 (Kanehisa et al., 2017). Modeling a small-sized network is a challenging task because a small number of molecular interactions limit the overall dynamic range of the signals (Tkačik et al., 2009; Azpeitia et al., 2020). Therefore, we discarded KEGG pathways having less than 30 edges from the interactome evaluations.

Among the selected interactomes, iRefWeb, HIPPIE, ConsensusPathDB, and STRING have edge confidence scores that are calculated with different scoring approaches. We applied an all-pair comparison of the given interactomes (***G, H***) with the Pearson correlation analysis on the confidence scores in the intersection of edge sets in interactome pairs (***E***_***G***_ ***∩E***_***H***_)

Biological networks follow the scale-free power law distribution, ***P(k) = k*** ^***-ɣ***^, where k is the degree of a node, and ***ɣ*** is the power coefficient (Barabási and Albert, 1995; Alm and Mack, 2016). To linearize the representation of both degree distribution and publication distribution, the logarithm of distribution was used as ***log(P(k)) = −ɣlog(k)*** *.* We collected the number of publications about each protein from UniProt. The correlation between the degree and the number of publications of the nodes was evaluated using the Pearson correlation test on a log scale.

### Network Reconstruction Methods

We used four reconstruction approaches, the shortest path, heat diffusion, PageRank, and PCSF. Selected interactomes are separately employed as the reference network, ***G(V, E, c(e)*)**, where ***V*** is the node set, ***E*** is the undirected edge set, and ***c(e)*** is the weight of an edge. These networks are weighted by confidence scores in the interactions, ***0≤c(e)≤1***. Network reconstruction algorithms infer the subnetwork, ***R(V***_***R***_ ***, E***_***R***_ ***)***, where ***V***_***R***_ ***⊆ V*** and ***E***_***R***_ ***⊆ E***, by connecting the seed node set, ***V***_***I***_ ***⊆ V***. The given node set is weighted with uniform ***1/|V***_***I***_ ***|*** where ***|V***_***I***_ ***|*** is the number of seed nodes, while the remaining node set is weighted as ***0***, so that ***w(v)*** can be defined for reconstruction algorithms.

#### All-Pairs Shortest Paths

We found out all shortest paths between each pair of nodes, ***u*** and ***v ∈ V***_***I***_ ***, u≠v***. When there are multiple shortest paths between u and v, we included all of them. Finally, we merged all shortest paths to obtain the final subnetwork. We did not put any edge weight–based filtering or path length threshold.

#### Personalized PageRank

The PageRank algorithm was normally designed for propagation in directed graphs. Personalized PageRank (PPR) is adapted to undirected graphs by converting each edge into both directed edges. The PageRank score of each node, ***p(v)***, in the reference interactome, ***G***, represents the probability of being at the node at a certain time step (***t***) that is calculated using the following iterative formula:

$$p_{t+1}\left(y\right)=\frac{1-\lambda }{N}+\lambda \sum_{x_{i}\to y}\frac{p_{t}\left(x_{i}\right)}{\deg\left(x_{i}\right)}\;$$ (2)

where Eq. 2 includes the probability of node ***y ∈ V*** that is calculated using the damping factor (***λ***) defining the probability of walking from neighbor nodes (***x***_***i***_) to ***y***, and ***N*** is the number of nodes (Page et al., 1998; Langville and Meyer, 2005). Initial probabilities of nodes were taken from ***w(v)***. We iterated Eq.2 100 times by default to obtain ***p(v)***.

#### Heat Diffusion

In the heat diffusion (HD), seed nodes having uniform heats prioritize their related nodes *via* heat transfer, which is formulated as follows:

$$p\left(v\right)=p_{0}{\left(I+\frac{-\alpha }{N}\text{L}\right)}^{N}$$ (3)

In Eq. 3, ***L = I –W***, where ***I*** represents an identity matrix and ***W = D*** ^***−1***^ ***A*** in which ***D*** and ***A*** are defined as the diagonal degree matrix and the adjacency matrix, respectively. ***p***_***0***_ is the initial heat vector in which nodes were weighted from ***w(v)***. ***N*** and ***α*** are, respectively, the number of iterations and the heat diffusion rate. ***N = 3*** is set as the default (Nitsch et al., 2010). At the end of heat diffusion, nodes have the diffused heat ***p(v)*** as the weight.

#### Edge Selection Over Flux Scores

Personalized PageRank with flux (PRF) and heat kernel diffusion with flux (HDF) are calculated over ***deg(v)***, which is defined as the number of interactions in ***G***, and node scores **0≤*p(v)*≤1**, which come from PPR or HD. In our study, unlike TieDie and HotNet with heat diffusion algorithms and flux on a random walk with restart, the threshold value is employed to eliminate uncritical nodes (Vandin et al., 2011; Creighton et al., 2013; Rubel and Ritz, 2020). The related nodes with ***p(v***_***i***_ ***)≥1/n*** where ***n*** is the number of nodes in the interactome are considered for subnetwork reconstruction. We calculated the directional flux scores ***f***_***u→t***_ using Eq. 4 where ***u, t ∈ V***, ***p(u)*** is the score that comes from PPR or HD, and ***deg(u)*** is the number of neighbors of node ***u***. Likewise, we calculated ***f***_***t→u***_ using Eq. 5. We determined the final flux of the edge as the minimum of ***f***_***u→t***_ and ***f***_***t→u***_ (Eq. 6).

$$f_{u\to t}\left(u,t\right)=\frac{p\left(u\right)\times c\left(e\right)}{deg\left(u\right)}$$ (4)

$$f_{t\to u}\left(t,u\right)=\frac{p\left(t\right)\times c\left(e\right)}{deg\left(t\right)}$$ (5)

$$f\left(e\right)=min\left(f_{u\to t}\left(u,t\right),\;f_{t\to u}\left(t,u\right)\right)$$ (6)

Edges are ranked from the highest flux score to the lowest by taking the negative logarithm of the flux. A total flux (***F***) is calculated among the related nodes as follows:

F=∑f(e) (7)

**0≤τ ≤ 1,** where **τ** is a flux threshold value that is the selection percentage of ***F***. Edges are selected by summing flux scores from the highest to the lowest until the targeted flux amount, ***τxF***. The edges having low flux scores are excluded from reconstructed subnetworks (Rubel and Ritz, 2020).

#### Prize-Collecting Steiner Forest

We used the PCSF algorithm implemented in Omics Integrator2. The seed nodes ***, v***_***i***_ ***∈ V***_***I***_, are weighted uniformly, and the edge costs are calculated using the cost function implemented in Omics Integrator 2 which combines the edge confidence score, *c*(*e*), and a penalty calculated from node degrees scaled with the *γ* parameter. If the reference interactome does not have confidence scores, ***c(e) = 1*** is uniformly defined. PCSF also penalizes the nodes based on their degrees (Tuncbag et al., 2016a). The new version, Omics Integrator 2, penalizes the edges based on the degrees of the node pair. The following function finds an optimum forest, ***F(V, E)***, by minimizing the objective function (Tuncbag et al., 2013):

$$f^{\prime }\left(F\right)\;=\sum \beta .p\left(v\right)+\sum cost\left(e\right)+\omega .\kappa$$ (8)

In Eq. 8, **κ** is the number of connected components, ***β*** controls the relative weight of the node prizes, and ***ω*** controls the cost of adding an additional tree to the solution network.

PCSF provides an optimum forest for each parameter set and an augmented forest which includes all the edges in the interactome that are present between the nodes in the optimal forest. We obtained the final reconstructed networks with the intersection of the optimal augmented forests that were generated using multiple parameter sets.

### Performance Analysis

NetPath is the curated human signaling pathway database that is composed of immune signaling pathways and cancer signaling pathways. In this study, 32 pathways in NetPath were used as a plausible dataset (Kandasamy et al., 2010). Since the computational cost of reconstruction was expensive for all pathways in NetPath with all parameter sets, first, optimum parameter sets were determined before performance analysis.

#### Parameter Tuning

Parameters of reconstruction algorithms were separately optimized for each reference interactome. Thus, Wnt, TCR, TNFα, and TGFβ pathways on NetPath were used for parameter selection. Nodes in each pathway were independently shuffled and split into five-fold. Each fold was, respectively, removed from the complete pathway node list, and network reconstruction was executed with the remaining folds. Parameters of reconstruction algorithms were separately tuned for each reference interactome to maximize the F1 score (Eq. 12). In the APSP, all identified shortest paths among seed node sets were inserted into a reconstructed pathway without any parameter tuning, so we do not adjust any parameter. We tuned the parameters in the given interval in Table 2 for PRF, HDF, and PCSF and for each reference interactome. Parameter sets of PRF and HDF were tuned in a two-dimensional grid *via* the mean of parameters that pooled the 10 highest F1 scores (Supplementary Figures 1, 2). In the PCSF, the union of all parameters that achieve the best coverage of the seed nodes, ***V***_***I***_, for each pathway was used as optimum parameter sets.

**TABLE 2:** Tuning ranges of parameter sets in PageRank flux (PRF), heat diffusion flux (HDF), and prize-collecting Steiner forest.

Reconstruction algorithm	Parameter	Range	Increment
PRF	Damping factor (*λ*)	0–1	0.05
	Flux threshold (***τ***)	0–1	0.05
HDF	Heat diffusion rate(***α***)	0–1	0.05
	Flux threshold (***τ***)	0–1	0.05
PCSF	Dummy edge weight (***ω***)	0–5	0.5
	Edge reliability (***β***)	0–5	0.5
	Degree penalty (***γ***)	0–10	0.5

#### The Calculation of Performance Scores

After tuning the parameters on four pathways, the remaining 28 pathways in NetPath, listed in Supplementary Table 1, were used for performance evaluation with five-fold cross-validation. We evaluated each reconstruction algorithm separately on each reference interactome by calculating the F1 score, Matthew’s correlation coefficient (MCC), recall and precision values, and false positive rate (FPR) in Eqs 9–13 as follows:

$$recall\left(TP,TN\right)=\frac{\left|TP\right|}{\left(\left|TP\right|+\left|FN\right|\right)}$$ (9)

$$precision\left(TP,FN\right)=\frac{\left|TP\right|}{\left(\left|TP\right|+\left|FP\right|\right)}$$ (10)

$$FPR\left(TP,FN\right)=\frac{\left|FP\right|}{\left(\left|FP\right|+\left|TN\right|\right)}$$ (11)

$$F1_{score}=\frac{2\times precision\;x\;recall}{precision+recall}$$ (12)

$$MCC\left(TP,TN,FP,FN\right)=\frac{\left(\left|TP\right|\times \left|TN\right|\right)-\left(\left|FP\right|\times \left|FN\right|\right)}{\sqrt{\left(\left|TP\right|+\left|FP\right|\right)\left(\left|TP\right|+\left|FN\right|\right)\left(\left|TN\right|+\left|FP\right|\right)\left(\left|TN\right|+\left|FN\right|\right)}}$$ (13)

Seed nodes were not counted in the performance calculation. However, all edges in the reconstructed network were used in the performance evaluation since interactions were not used in the initial input. For a given reference in interactome **G(*V, E*)** and an seed node set ***(V***_***I***_ ***)*** from a pathway ***T(V***_***T***_ ***, E***_***T***_ ***)***, a network is reconstructed, ***R(V***_***R***_ ***, E***_***R***_ ***)***, using the listed methods, where ***V***_***T;***_, ***V***_***R***_ and ***V***_***I***_ ***⊆ V***, and ***E***_***T***_ and ***E***_***R***_ ***⊆ E***. Node-level true positives ***(TP***_***V***_) and edge-level true positives (***TP***_***E***_) are obtained from | ***V***_***R***_ ***⋂ V***_***T***_ | and | ***E***_***R***_ ***⋂ E***_***T***_ |, respectively. Node-level true negatives ***(TN***_***v***_) and edge-level true negatives ***(TN***_***E***_) are obtained from *|* ***V \ (V***_***R***_ ***⋃ V***_***T***_ *)| and |* ***E \ (E***_***R***_ ***⋃ E***_***T***_ *)|*, respectively. False positives ***FP***_***V***_ and ***FP***_***E***_ are equal to | ***V***_***R***_ ***\ V***_***T***_ | and | ***E***_***R***_ ***\ E***_***T***_ |, respectively. False negatives ***FN***_***V***_ and ***FN***_***E***_, are equal to | ***V***_***T***_ ***\ V***_***R***_ | and | ***E***_***T***_ ***\ E***_***R***_ |, respectively.

We performed principal component analysis (PCA) to figure out critical scores that explain the highest variance across all pathways. We statistically assessed overall performance data including both edge- and node-based scores by individually grouping reference interactomes and reconstruction methods.

### Data Availability Statement

Codes and datasets used for this study are publicly available at the online repository https://github.com/metunetlab/Interactome_Network_Reconstruction_Assessment_2021. We downloaded NetPath, http://netpath.org/browse, and PathwayCommons, https://www.pathwaycommons.org/archives/PC2/v12/PathwayCommons12.All.hgnc.txt.gz, iRefWeb, http://wodaklab.org/iRefWeb/search/index, HIPPIE, http://cbdm-01.zdv.uni-mainz.de/∼mschaefer/hippie/download.php, STRING, https://string-db.org/cgi/download, ConsensusPathDB, http://cpdb.molgen.mpg.de/, Reference Human Proteome from UniProtDB, https://www.uniprot.org/, using the query https://www.uniprot.org/uniprot/?query=proteome:UP000005640%20reviewed:yes, INSIDER, http://interactomeinsider.yulab.org/downloads.html, and KEGG, https://www.kegg.jp/kegg/download/ and http://rest.kegg.jp/get/+’pathwayid’+'/kgml'. OmniPath and the signaling pathways in Glioblastoma (WP2261) were retrieved from WikiPathway using Cytoscape 3.8.0.

## Results

### Systematic Evaluation of Reference Human Interactomes

**FIGURE 2:**
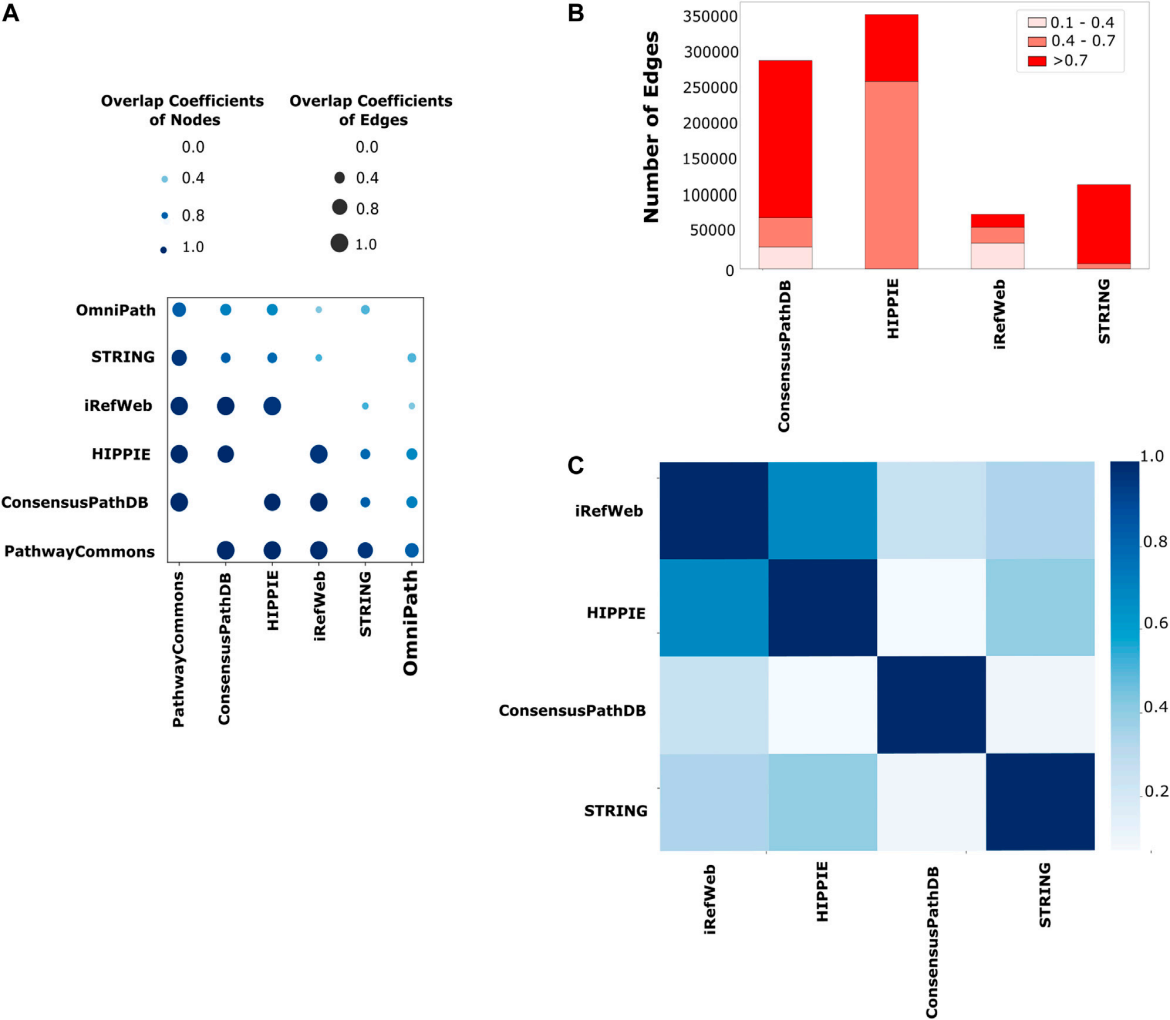
Comparison of the reference interactomes. **(A)** Node- and edge-level commonalities between different interactomes; the node overlap score is displayed on a light-to-dark blue-color scale, while the edge similarity score is shown on a size scale where the higher similarity is represented with the larger circle. **(B)** Scores are categorized as low confidence between 0.1 and 0.4, medium confidence between 0.4 and 0.7, and high confidence between 0.7 and 1.0 for each interactome. PathwayCommons and OmniPath are not demonstrated here due to the lack of a confidence score for their edges. Edges with low confidence scores are mostly seen in iRefWeb and small portions of edges in ConsensusPathDB have low confidence scores, while low confidence edges are not seen in the filtered STRING and HIPPIE. **(C)** Different strategies on confidence score calculation are used in interactomes. Using their common edges, the correlation coefficients among their confidence scores over interactomes are demonstrated in the heatmap. The highest correlation between HIPPIE and iRefWeb is seen with the darkest blue. The same confidence score calculation strategy, MI scoring, is used in both interactomes.

Network reconstruction algorithms are highly dependent on the quality and coverage of the reference interactome. Therefore, we systematically explored the properties of iRefWeb, PathwayCommons, HIPPIE, ConsensusPathDB, OmniPath, and STRING databases. Among them, iRefWeb, HIPPIE, ConsensusPathDB, and STRING provide the measure of confidence in interactions as scores. First, we compared the pairs of interactomes to determine how similar they are in terms of their node and edge sets. PathwayCommons is the largest network in size, so it has the highest fraction of node and edge overlap compared to all other interactomes. iRefWeb, PathwayCommons, HIPPIE, and ConsensusPathDB are the most similar interactomes to each other based on the node and edge overlaps (Figure 2A). On the other hand, STRING and OmniPath have fewer common nodes and edges with other interactomes. We need to note that the raw data in STRING contain more than one million interactions in human interactomes, and we used only the experimental and database interactions which resulted in a relatively small-sized interactome with medium or high confidence edges. Before using the network reconstruction algorithms, obtaining the reference interactome with measurements of interaction confidence is fundamental to decreasing the impact of the false positives. Because network reconstruction algorithms leverage the edge confidence scores and the topology of the reference interactomes during the propagation or optimization, confidence scores may substantially affect the accuracy of the resulting network. Even two topologically equivalent interactomes may produce different subnetworks as a result of network reconstruction if their confidence score distributions are different from each other. In Figure 2B, the number of edges in each reference interactome is shown, which are categorized as low, medium, and high confidence edges based on the interaction scores. ConsensusPathDB contains predominantly high confidence interactions, while HIPPIE and iRefWeb interactions are accumulated in medium and low confidence intervals. HIPPIE and iRefWeb use MINT-inspired (MI) confidence score calculation, while ConsensusPathDB uses the IntScore tool (Braun et al., 2009; Turner et al., 2010; Kamburov et al., 2011; Kamburov et al., 2012; Turinsky et al., 2011; Schaefer et al., 2012; Alanis-Lobato et al., 2017). We recalculated the confidence scores in STRING by considering only the experiment and database scores. PathwayCommons and OmniPath do not provide confidence scores. Edge confidence scores can be computed in various ways. Different scoring schemes lead to variation in the confidence score distributions across the interactomes. As expected, the correlation of confidence scores between HIPPIE and iRefWeb is the highest (*r* = 0.67, *p* < 0.01) because both use MI-Score. The correlation between confidence scores in iRefWeb and ConsensusPathDB is very low (*r* = 0.25, *p* < 0.01) (Figure 2C) because ConsensusPathDB uses a different scoring scheme, IntScore. While MI-Score considers homologous interactions, the detection method, and the number of publications about the interactions, IntScore includes topological properties, literature evidence, and similarities in annotation of proteins.

Confidence scores do not completely solve the bias in the interactomes despite being a powerful measurement to filter out false positives. Therefore, we additionally analyzed the interactomes based on the bias toward well-studied proteins using different features, namely, the number of publications about the proteins, coverage of the cancer driver genes, and the number of interactions having structural details. Well-studied proteins, such as TP53 and EGFR, have hundreds of high confidence interactions in the interactomes (Schaefer et al., 2015; Chen et al., 2018; Porras et al., 2020). Indeed, there is a trade-off between the interaction confidence scores of certain proteins and systematic study bias. We used the number of publications and the degree centrality of proteins in each reference interactome to explore if highly connected proteins are also well-studied ones.

**FIGURE 3:**
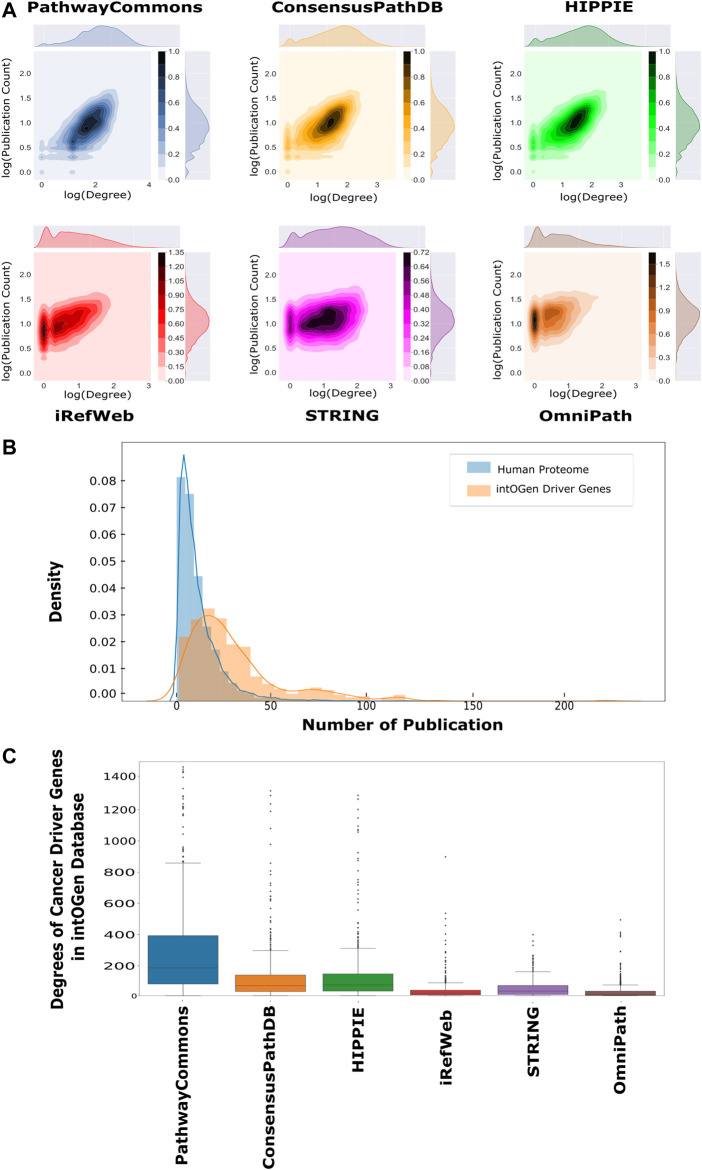
Correlation between publication counts and degrees over interactomes. **(A)** Log–log scale joint graphs of publication distribution and degree distribution for each interactome were drawn since both follow a power-law distribution. While all interactomes have a positive correlation between the degree and publication number, PathwayCommons, HIPPIE, ConsensusPath, and iREF have well-studied hubs. On the other hand, hubs in iRefWeb and OmniPath are not composed of relatively well-studied proteins (p-values <0.001 and r_PathwayCommons_ = 0.622, r_ConsensusPathDB_ = 0.556, r_HIPPIE_ = 0.614, r_iRefWeb_ = 0.508, r_STRING_ = 0.250 and r_OmniPath_ = 0.400). **(B)** Distributions of the number of publications and cancer driver genes in the intOGen database are shown, respectively, in blue and orange. The probability of well-studied cancer driver genes (CDGs) is higher than the probability of well-studied proteins. **(C)** Driver gene degrees in the interactomes are demonstrated in the boxplot in which driver genes in PathwayCommons have more connection than other interactomes. OmniPath and iRefWeb do not have highly connected driver genes as many as ConsensusPath, HIPPIE, STRING, and PathwayCommons.

Each analyzed interactome is a scale-free network so that their degree distributions follow the power law (Supplementary Figure 3) (Barabási and Albert, 1995; Vidal et al., 2011). The number of publications about proteins follows the power law distribution as their degree distribution (Supplementary Figure 4). Thus, the number of publications and degrees were analyzed using log-based values to find out their correlation. The number of publications and the degrees of proteins are positively correlated in all interactomes (Figure 3A **)**. We observed the highest correlation in PathwayCommons (*r* = 0.62, *p* < 0.01) and HIPPIE (*r* = 0.61, *p* < 0.01), which implies the bias toward well-studied proteins in these interactomes. iRefWeb, STRING, and OmniPath have moderate correlation between the degree and the number of publications, which implies relatively less biased interactomes (Supplementary Table 2). We note that this comparison is performed on the whole interactome without any confidence score–based filtering, except STRING. We expect that if only the high or medium confidence interactions in other interactomes would be considered, the correlations may be dramatically reduced and the bias toward well-studied proteins may be dumped. Reconstruction algorithms are also adapted to overcome this inherent bias toward the nodes and edges in interactomes. For example, heat diffusion and random walk, together with the edge flux calculation, use node degrees for normalization, while PCSF penalizes highly connected proteins (Creixell et al., 2015; Tuncbag et al., 2016a; Rubel and Ritz, 2020). In this way, false-positive edges belonging to hub nodes are excluded from the final subnetwork.

One application area of network reconstruction algorithms is the discovery of disease-associated pathways, especially in cancer, by inferring the seed proteins/genes. The resulting networks are used for patient stratification, biomarker discovery, or the analysis of drug mechanisms of action (Mo et al., 2018; Huang et al., 2019; Koh et al., 2019; Wang et al., 2021). Therefore, we searched for the coverage of the cancer driver genes (CDGs) in each interactome. CDGs provide growth advantage to the tumor cells and alter signaling pathways. Additionally, CDGs are important markers in tumor stratification, characterization, and drug development (Waks et al., 2016; Bailey et al., 2018; Zsákai et al., 2019). We obtained the list of CDGs from the intOGen database (Martínez-Jiménez et al., 2020). We found that significantly more publications are present for CDGs than for the rest of the proteomes, as shown in Figure 3B (*p* < 0.01). The presence of driver genes and their edges help in accurately reconstructing the driver pathways in cancer. All analyzed interactomes are highly inclusive of driver genes, especially PathwayCommons, ConsensusPathDB, and HIPPIE (Supplementary Figure 5). However, the degrees of CDGs in the PathwayCommons interactome are significantly higher than others (Figure 3C **)**.

**FIGURE 4:**
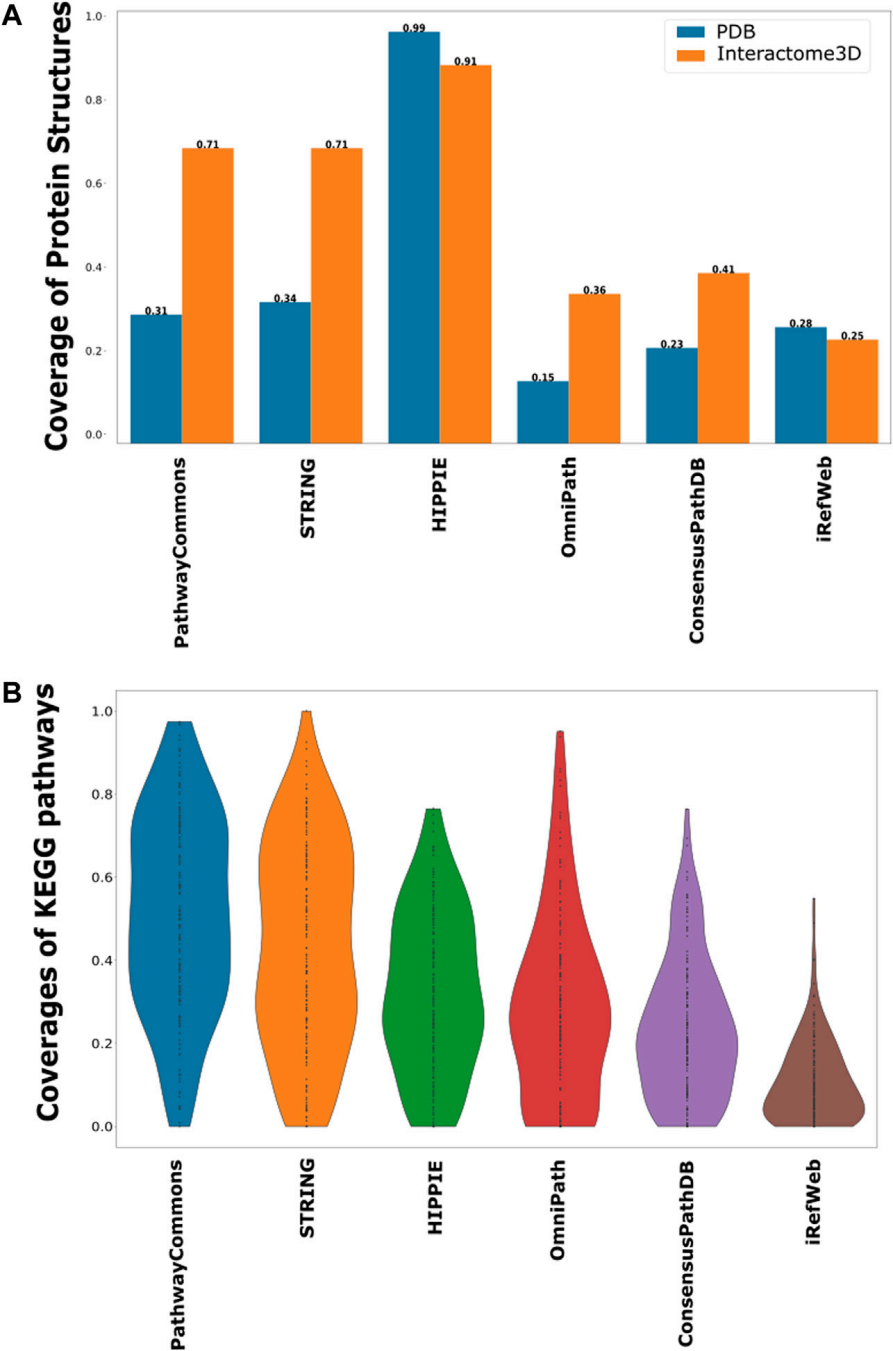
Coverage of structurally known interactions and pathway interactions in each interactome. **(A)** Structural information is demonstrated in two groups, as known interactions in PDB in blue and predicted interactions in Interactome3D in orange. **(B)** Overlaps between the interactions in KEGG pathways and each interactome are shown as a violin-plot.

In terms of protein interactions, the most accurate and confident interactions can be caught by their structural identification. Structures of protein–protein complexes uncover the binding sites, domain contacts, and many more (Schmidt et al., 2014; Nero et al., 2018; Hicks et al., 2019). The only drawback is the availability of limited structural data. Despite the exponential increase in PDB with the help of the X-ray, CryoEM, and NMR techniques, the number of protein complexes can still only cover around 16% of the whole interactome (Berman et al., 2000; Mosca et al., 2013; Venko et al., 2017). Many structure-based predictive approaches are also employed to accurately identify protein–protein interactions. Therefore, we further analyzed each interactome based on the representation of structurally annotated interactions. For this purpose, we used the complexes in PDB and Interactome3D. We found that HIPPIE has the highest coverage of structurally known protein–protein interactions (Figure 4A). HIPPIE is followed by PathwayCommons and ConsensusPathDB. iRefWeb, OmniPath, and the filtered STRING interactome have the lowest coverages.

**FIGURE 5:**
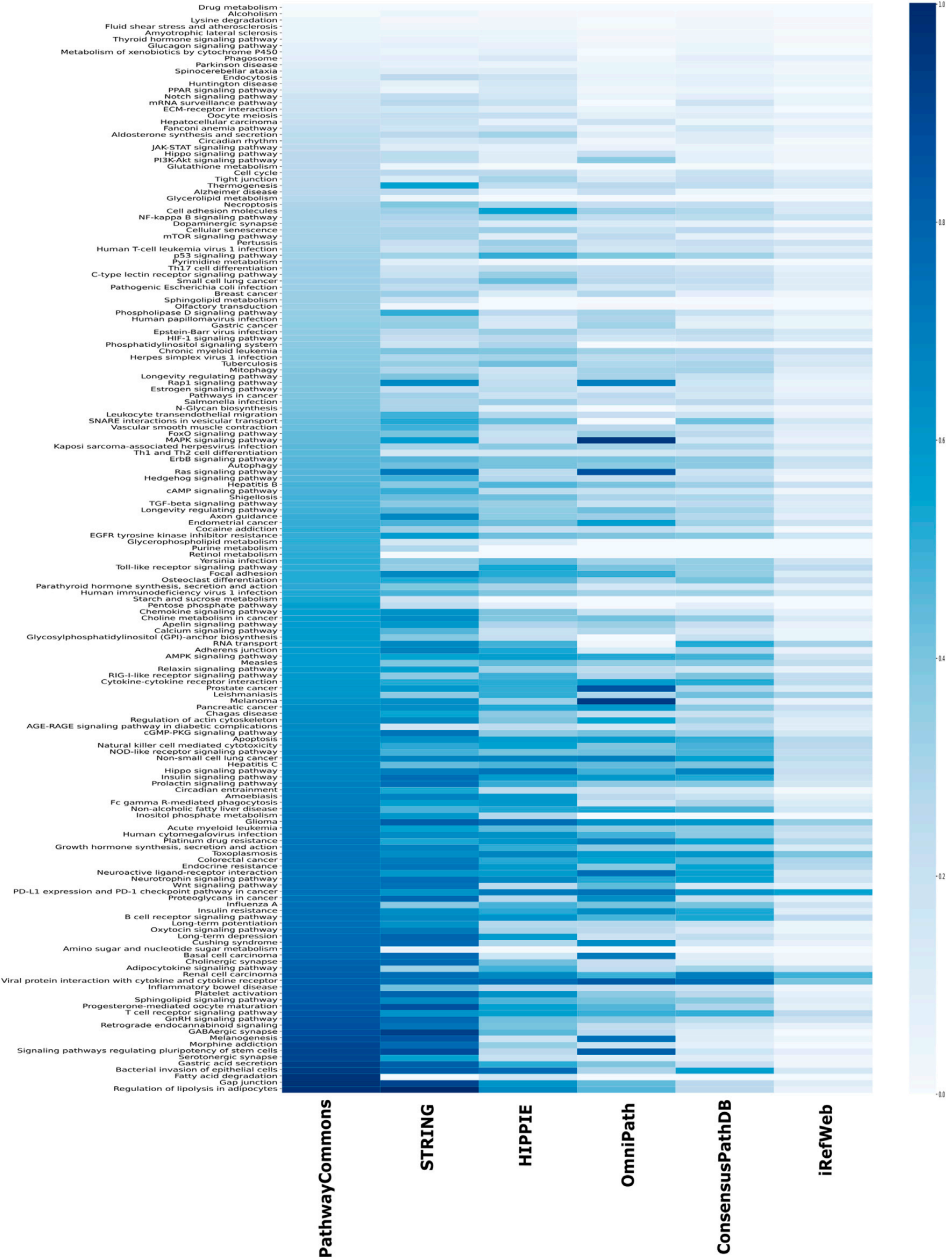
Overlap between each pathway in KEGG and each interactome. The sorted coverage of interactomes for 171 KEGG pathways is shown as a heatmap.

Another source of confident interactions is the curated pathways, despite being incomplete. Generated subnetworks are required to be biologically meaningful so that their downstream analysis can sign proper biological functions (Vidal et al., 2011; Sevimoglu and Arga, 2014). Therefore, we explored the coverage of interactomes based on the curated pathways retrieved from KEGG, which is one of the most frequently used databases for pathway annotations. We found that KEGG pathways are relatively less represented in iRefWeb, while PathwayCommons and filtered STRING highly covered them (Figure 4B). We need to note that some individual pathways are better covered in some interactomes although their overall coverage is relatively low (Figure 5). For example, the MAPK and RAS signaling pathways are better represented in OmniPath, although OmniPath has a moderate coverage of all pathways. Individual pathway coverage of each interactome is listed in Supplementary Table.

### Performance of Network Reconstruction Algorithms

As evidenced in detail, each interactome has its own strengths and weaknesses. These properties have a direct effect on the performance of network reconstruction algorithms. Therefore, we used each interactome as the reference for each network reconstruction algorithm to monitor the variance in the performance. We used four well-established network reconstruction algorithms, the all-pair shortest paths (APSP), personalized PageRank with flux (PRF), heat diffusion with flux (HDF), and prize-collecting Steiner forest (PCSF) algorithms, to evaluate their performance on the gold standard dataset of 32 curated pathways retrieved from NetPath. Four pathways are used for parameter tuning, and the rest (28 pathways) is used for performance evaluation.

**FIGURE 6:**
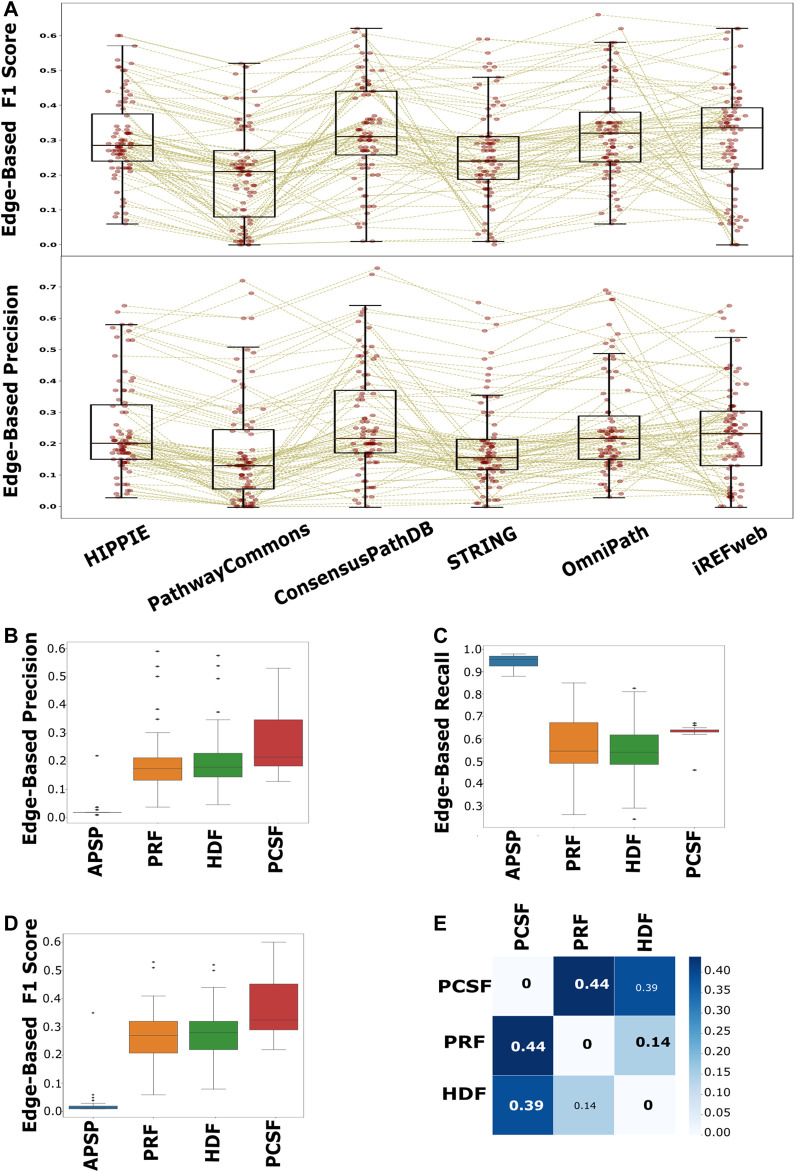
Performance evaluation of each interactome and method in pathway reconstruction. **(A)** Boxplot of edge-based precision and F1 scores, for each interactome, shows that PathwayCommons and STRING are significantly lower than HIPPIE, ConsensusPathDB, OmniPath, and iRefWeb, while there is not any distinct difference among HIPPIE, ConsensusPathDB, OmniPath, and iRefWeb. The performance values for each reconstructed network is represented with red points in the boxplots. Brown lines connect the performance scores of the same pathway across the interactomes. **(B)** Edge-based precision, **(C)** edge-based recall, and **(D)** edge-based F1 scores are separately demonstrated for reconstruction algorithms. **(E)** HDF, PRF, and PCSF were compared in terms of the reconstructed pathways. The heatmap shows that the reconstructed pathways by PCSF are different, having %44 and %39 different edges, respectively, than the ones reconstructed by PRF and HDF.

We collected both node- and edge-level performance metrics for each pair of interactomes and reconstruction methods on each pathway. We found that node-level performance is relatively more robust to different interactomes or different pathways in each approach than the edge-level performance. The largest variation is in the edge-level F1 scores, in that the balance between the recall and precision values is highly variable across pathways and interactomes (Supplementary Figure 6). The F1 scores (*p* < 0.001) and precision (*p* < 0.001) scores of the reconstructed pathways that are inferred from PathwayCommons are mostly lower than the scores of HIPPIE, ConsensusPathDB, OmniPath, and iRefWeb (Figure 6A). The second highest variation is in the edge-level MCC, used for binary classification over imbalanced data (Boughorbel et al., 2017; Magnano and Gitter, 2021). This result implies that the algorithms do not perform well with a relatively very large reference interactome because of the potential dominance of false positives over the true-positive interactions. Based on the F1 score and the precision value, we did not find a significant difference in performance when HIPPIE, ConsensusPathDB, OmniPath, or iRefWeb interactomes are used. Therefore, we continued with HIPPIE as a reference interactome for further assessments since it has the most balanced features based on the comparison in the previous part, including coverage of structurally known interactions. The comparison of edge-based performance scores showed that APSP significantly has the lowest precision values (*p* < 0.001) and the highest recall values among all reconstruction approaches when the performance across all pathways is evaluated. There is no significant difference in precision values between HDF, PRF, and PCSF (Figure 6B). The recall values of the reconstructed pathways do not significantly differ between HDF and PRF, while PCSF (*p* < 0.001) has significantly higher recall scores (*p* < 0.001) than HDF and PRF (Figure 6C **)**. The trade-off between the precision and recall scores can be noticed in the results of reconstruction methods. Insertion of all shortest paths between the seed nodes in the APSP algorithm causes both the reduction in precision values and the increase in recall values. The significantly high FPR in APSP (*p* < 0.001) indicates that false-positive edges dominate the true-positive edges (Supplementary Figure 7). Therefore, F1 scores of the APSP-reconstructed pathways are significantly lower than those of other methods (*p* < 0.001) (Figure 6D). On the other hand, PCSF-reconstructed pathways have moderately high recall and precision scores and the highest F1 score by a considerable margin, optimizing the trade-off between the precision and recall values. Interestingly, the interval of recall scores in the reconstructed pathways in PCSF is not variable in a wide interval as in other methods; rather, it fluctuates around 0.65. The PCSF approach gives an optimum forest as an output together with an augmented forest which includes all the edges in the interactome that are present between the nodes in the optimal forest. We obtained the final network of PCSF by taking the intersection of augmented forests from multiple parameters. In this way, adding an edge to the final network was made very stringent. We computed the Jaccard similarity matrix among HDF, PRF, and PCSF to demonstrate the variation on the edge-level performance in the reconstructed pathways (Figure 6E; Ricotta et al., 2016). PCSF penalizes highly connected nodes, which reduces the dominance of well-studied or highly connected nodes in the reconstructed networks. In this way, important but low-degree nodes are also successfully included in the reconstructed pathways. As a result, PCSF has balanced precision and recall values, and its reconstructed pathways have the highest dissimilarity compared to the reconstructed pathways from other methods. Overall, the performance of the algorithms is highly affected by the parameter selection along with the used background interactome. To illustrate the reconstructed networks intuitively and to distinguish their commonalities and differences for each algorithm, we selected two case studies; one is selected from the NetPath database and the other is selected from WikiPathways.

### Case Studies: Reconstruction of the Notch Pathway and Glioblastoma Disease Pathway

**FIGURE 7:**
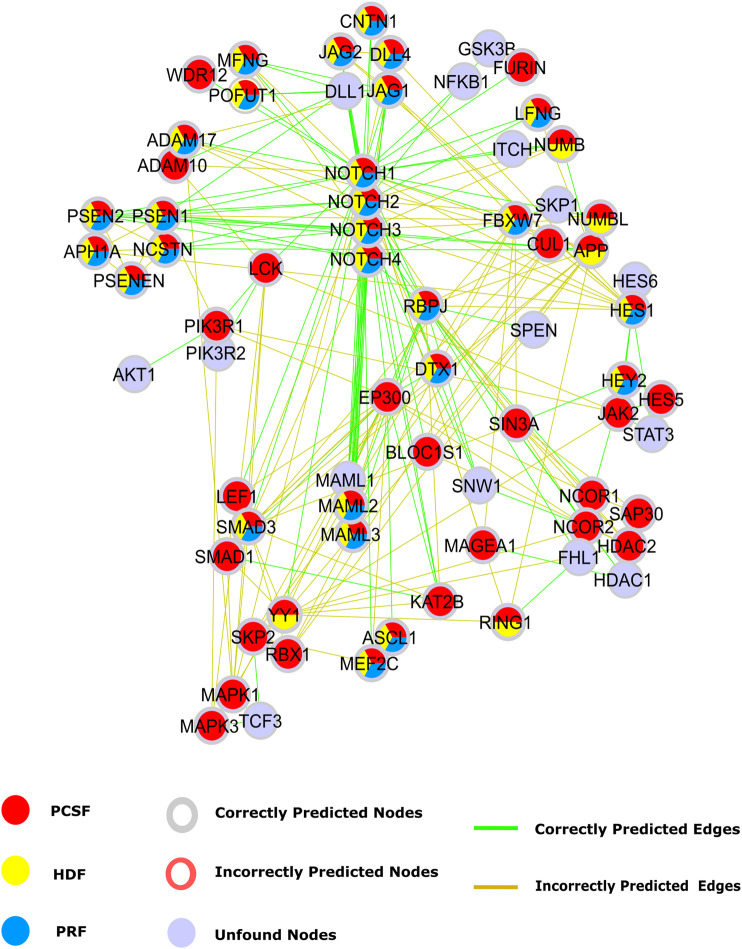
Reconstructed Notch pathway. Nodes that are present in the pathway, but are not found by any algorithms are colored light blue. Nodes that are found by PCSF, PRF, and HDF are colored red, yellow, and cyan, respectively. Green edges are present in Notch pathway in NetPath, while incorrectly included edges by any algorithm are shown in brown.

Our first case study is the Notch signaling pathway to intuitively illustrate the performance of each approach. The Notch signaling pathway plays a critical role in cell fate determination by regulating differentiation, apoptosis, proliferation, and morphogenesis. Its signaling cascades are associated with many human cancers (Sjölund et al., 2005; Bazzoni and Bentivegna, 2019; Guo et al., 2019). The APSP method recovers many true-positive edges, but it also introduces many false positives in the Notch pathway (Supplementary Figure 7). Therefore, only PRF, HDF, and PCSF results inferred from a set of seeds selected from the Notch pathway are illustrated in Figure 7. Notch receptors are single-pass transmembrane proteins, receiving signals from transmembrane ligands such as JAG1, JAG2, DLL1, and DLL4. The given protein list includes Notch receptors and CNTN1, JAG2, and DLL4. All reconstruction algorithms successfully identified JAG1 and the interaction between Notch receptors and their ligands except for DLL. True-positive nodes having a low degree in the reference interactome were caught better by PCSF than by PRF and HDF. Additionally, PCSF accurately included nodes such as CNTN1, WDR12, LEF1, RBX1, SIN3A, and many other true positives in the final reconstructed network. Although PCSF performs well in recovering low-degree nodes, it could not include some other nodes such as AKT1, SKP1, SPEN, and TCF3 in the pathway. PCSF successfully found the interactions between Furin–Notch receptors that regulate the Notch pathway in cancer progression where Furin, a low-degree ligand, generates biologically active heterodimer receptors (Qiu et al., 2015). On the other hand, PCSF fails to construct the interactions including low-degree nodes such as JAK2 and WDR12. HDF and PRF mostly reveal the interaction between high-degree nodes such as MAML1 and Notch receptors since the heat diffusion and the PageRank algorithm tend to give high scores to these nodes.

The Notch pathway has cross talk with other critical pathways in cancer such as the PI3K-AKT-mTOR and JAK-STAT signaling pathways (Chan et al., 2007; Hillmann and Fabbro, 2019). The cross talk is mediated by the nodes with low-degree and high betweenness centrality in the reference interactome such as PIK3R1, LCK, and JAK2. Although we could reveal these intermediate nodes that are important in cross talk between multiple pathways with PCSF, we could not achieve the same performance in the added edges. Despite correctly identifying PIK3R1 interaction with Notch1 and LCK, interactions with PIK3R2 and AKT were not found. In the JAK-STAT and Notch pathway cross talk (Rawlings et al., 2004; Liu et al., 2010), we accurately found intermediate nodes such as JAK2, HES1, and HES5, but we failed in recovering their interactions with STAT3 in the PCSF-reconstructed pathway.

**FIGURE 8:**
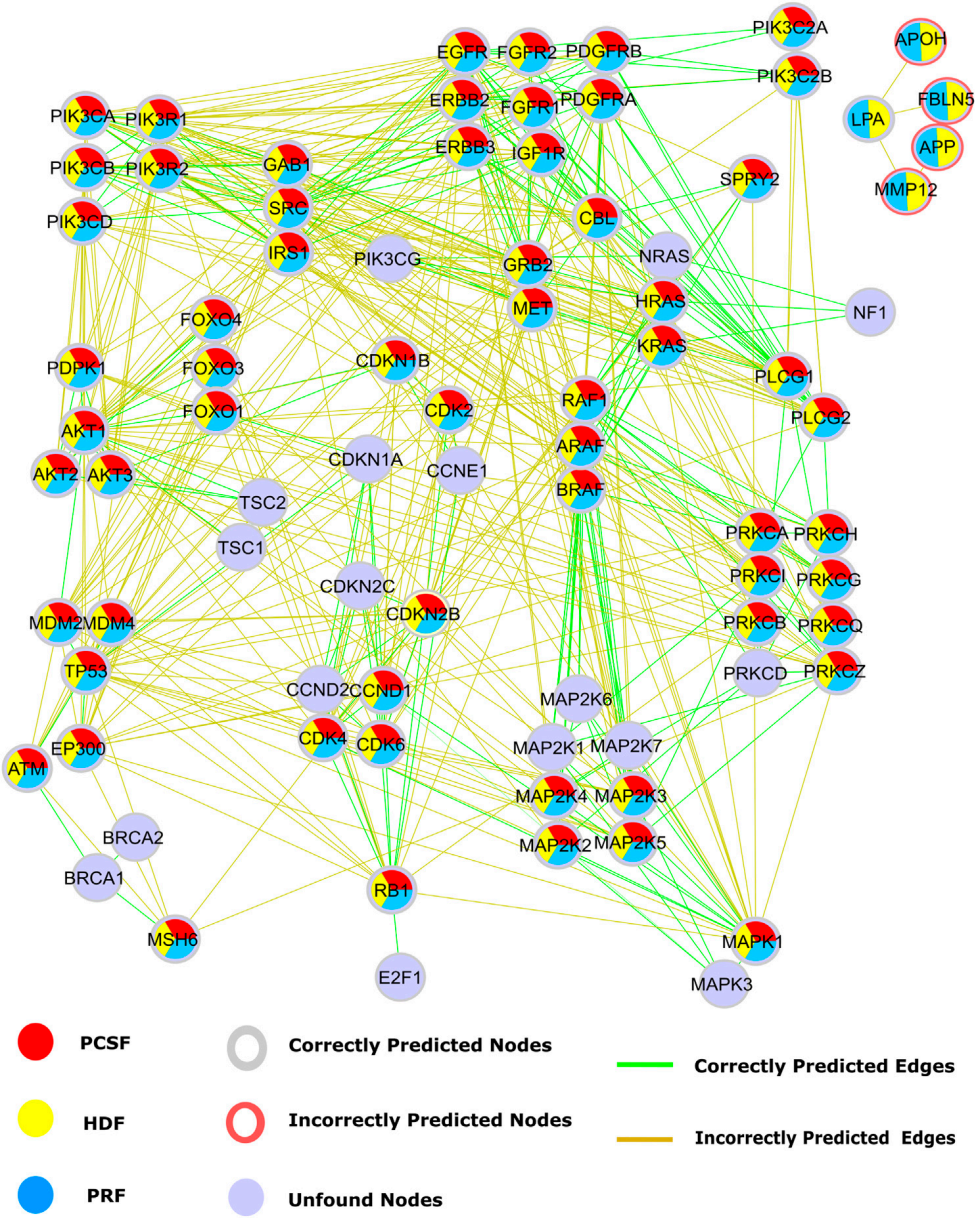
Reconstructed signaling pathways in glioblastoma (WP2261) are predicted by PCSF are red, PRF are yellow, HDF are cyan, and nodes that are not found by any algorithm are colored light blue. Additionally, red rings represent the nodes that are incorrectly propagated. Green edges are the correct ones that are present in GBM pathway, while incorrect edges included by any algorithm are colored brown.

Our second case study is the glioblastoma (GBM) disease pathway. Disease-related pathways are mostly composed of multiple signaling pathways. GBM is the most aggressive type of brain cancer. Multiple signaling pathways such as the PI3K/AKT/mTOR, EGFR/RAS/MAPK, P53, and RB pathways have abnormal activity in GBM tumors (Ohgaki and Kleihues, 2007). Disease-related pathways are mostly composed of multiple signaling pathways. The presence of cross talk *via* intermediate molecules is the reason why multiple pathways are related to a disease. In this regard, signaling pathways in GBM, retrieved from WikiPathways, were reconstructed by multiple algorithms using HIPPIE as the reference interactome. Multiple signaling pathways such as the PI3K/AKT/mTOR, EGFR/RAS/MAPK, P53, and RB pathways are associated with GBM. Alterations on these pathways may lead to more aggressive and invasive phenotype by disturbing DNA repair, apoptosis, and G1/S progression and enhancing cell cycle progression and cell migration (Ohgaki and Kleihues, 2007). Some nodes such as PIK3CG and CDK1NA and their interactions, mediating the cross talk between multiple pathways, were not efficiently revealed by reconstruction algorithms. CDKN1A is responsible for the inhibition of the RB signaling pathway by transducing signals coming from the PI3K/AKT/mTOR pathway. Even though the reconstructed subnetwork recovers the RB signaling pathway, all four algorithms failed in reconstructing the edges connecting two signaling pathways (Figure 8). Thus, these algorithms are good at revealing the mediator nodes in cross talk between pathways, but they fail in revealing the connection between them. The HDF and PRF methods ranked some nodes as important, such as APOH, FBLN5, AFP, and MMP12. Although these proteins are not present in the studied pathway, their association with GBM was previously discovered in transcriptomic or proteomic studies (Varma Polisetty et al., 2012; Kros et al., 2015; Trojan et al., 2020).

## Discussion

In this study, we comprehensively explored the properties of interactomes from seven sources and the performance of four network reconstruction algorithms on known pathways. Our comparison reveals that PathwayCommons, having the highest number of nodes and edges, has the highest coverage of nodes and edges across all interactomes, including CDGs, and known pathways. However, precision values of the reconstruction methods are significantly lower than the others when PathwayCommons is used as the reference interactome. We did not observe a significant difference in recall values among all interactomes. The significant correlation between the degree and the number of publications of the nodes in PathwayCommons shows a bias toward well-studied proteins. Interestingly, although HIPPIE and ConsensusPathDB have a similar bias, the precision of the algorithms on these interactomes is better than that of PathwayCommons. These results imply that HIPPIE and ConsensusPathDB have a good balance in down-weighting the false positives and preserving high confidence edges.

The results of the different network reconstruction algorithms may include disjoint edges. The highest recall scores in APSP come along with the highest FPR score because the APSP algorithm adds many false-positive edges besides the true positives. Some studies, such as PathLinker (Ritz et al., 2016), use a distance threshold during shortest path calculation, a limited number of shortest paths between the source and the target, or additional data including orientation of the signal from the receptors to the transcription factors so that the false positive rate is controlled. We need to note that we did not apply any distance-based threshold, additional data, or refinement in the APSP algorithm. Thus, F1 scores and precision scores are extremely low in APSP. On the other hand, PRF, HDF, and PCSF have similar performances of false positive and true positive edges. PCSF has the highest F1 score compared to PRF and HDF. Interactomes are imbalanced datasets where true-negative edges are significantly more than true-positive edges. Naturally, the precision scores seem relatively low in the pathways formed by our algorithms since the FPR gets higher in such imbalanced datasets. The reconstructed Notch pathway shows that PCSF is better at finding weakly connected nodes. However, PCSF does not perform well in revealing the intermediate nodes and their edges achieving the cross talk between the Notch pathway and the PI3K-AKT-mTOR and JAK-STAT signaling pathways. Moreover, the intermediate nodes that links signaling pathways in GBM cannot construct completely true edges. In our study, the nodes are proteins; however, pathways may include small molecules and non-peptide nodes. Therefore, the reconstruction algorithms probably add false edges to include true terminals. The lack of some nodes in reference interactomes may be one of the reasons for the low precision scores.

Network reconstruction algorithms are highly dependent on topological properties and edge weights of the reference interactomes (Janjić and Pržulj, 2017; Liu et al., 2017). Among the evaluated approaches, the highest recall values are achieved by using the APSP algorithm together with the lowest precision values. The APSP algorithm adds many false-positive edges, besides the true positives. On the other hand, PRF, HDF, and PCSF have similar performances, while PCSF has a higher F1 score than PRF and HDF. High recall scores together with low precision scores are the result of the unbalanced data where the number of edges in the target pathway is dramatically lower than that in the rest of the interactome (Saito and Rehmsmeier, 2015). The low precision score with the moderate recall score is common among reconstruction algorithms of human signaling networks (Atias and Sharan, 2011; Ritz et al., 2016; Grimes et al., 2019). Additionally, edge-based performances of reconstruction algorithms are not as good as their node-based performance. We also observe a similar pattern of performances in our evaluation.

In a recent study, the performance of flux algorithms was shown to exceed the performance of PCSF with default parameters (Rubel and Ritz, 2020). However, the selected set of parameters significantly affects the performance of reconstruction algorithms, especially in PCSF. Automating parameter tuning that considers topological properties of reconstructed subnetworks can improve the performance (Magnano and Gitter, 2021). Therefore, in this study, we reconstructed pathways by extensively tuning the parameter set, followed by merging multiple optimal forests to reach the best performance. Parameter sets of other reconstruction algorithms were also tuned to find the optimum parameters. We can explain the overperformance of PCSF compared to other methods with detailed parameter tuning and considering multiple optimal solutions.

Several methods use topological properties of reference interactomes to predict new links and to filter out false-positive interactions (Cannistraci et al., 2013; Lei and Ruan, 2013; Hulovatyy et al., 2014; Alkan and Erten, 2017). Additionally, functional annotations, protein structures, and domain–domain interactions were also used to identify missing protein associations (Singh et al., 2006; Segura et al., 2015; Yerneni et al., 2018; Ietswaart et al., 2021). We need to note that we did not use the methods that modify the underlying interactome (Alanis-Lobato et al., 2018) and the methods that construct regulatory networks (Madar et al., 2009; Fontaine et al., 2011; Lachmann et al., 2016) in our evaluation. The performance of the APSP, HDF, PRF, and PCSF algorithms may change upon any modification or refinement of the reference interactomes. These reference interactomes are undirected graphs, but signaling pathways are intrinsically directed graphs. Indeed, the directionality of the edges can be incorporated either with the known or with the predicted ones. Orientation of the reconstructed networks can improve the mechanistic understanding of biological pathways. Therefore, using a directed reference interactome can boost the performance of each algorithm. Finally, biomolecular interactions are temporally and spatially diverse. Interactomes are incomplete sets of interactions, and the time dimension is not considered in our evaluation. Subnetwork reconstruction algorithms may be improved in the future to include biological annotations and temporal and spatial interactions of proteins.

## Data Availability

The datasets presented in this study can be found in online repositories. The names of the repository/repositories and accession number(s) can be found in the article/Supplementary Material.

## Abbreviations

APSP, all-pairs shortest paths; CDGs, cancer driver genes; FPR, false positive rate; HD, heat diffusion; HDF, heat kernel diffusion with flux; MCC, Matthew’s correlation coefficient; MI, MINT inspired; PCA, principal component analysis; PCSF, prize-collecting Steiner forest; PPR, personalized PageRank; PRF, personalized PageRank with flux
