# Supplementary material for: Performance Assessment of the Network Reconstruction Approaches on Various Interactomes
Source: Front Mol Biosci. 2021 Oct 5;8:666705. doi: 10.3389/fmolb.2021.666705 (PMC8523993; doi:10.3389/fmolb.2021.666705)
Supplement: Supplementary file 1 [file DataSheet1.pdf]

Supplementary Information: A critical assessment of the performance of network reconstruction approaches based on interactome data sets

Sampling Pathways in Performance Metrics		
**Pathway Name**	**Node Size**	**Edge Size**
Alpha6Beta4Integrin	66	116
AndrogenReceptor	165	251
BCR	137	261
BDNF	72	76
CRH	24	27
EGFR1	231	756
FSH	19	18
Hedgehog	36	64
ID	27	51
IL1	43	93
IL11	16	22
IL2	67	139
IL3	70	97
IL4	57	91
IL5	30	36
IL6	53	83
IL7	18	28
IL9	13	15
KitReceptor	76	109
Leptin	55	74
Notch	74	154
OncostatinM	37	42
Prolactin	68	103
RAGE	23	25
RANKL	57	76
TSH	48	47
TSLP	7	7
TWEAK	17	15
**Pathways used in the parameter tuning**		
**Pathway Name**	**Node Size**	**Edge Size**
TCR	154	271
TGFbetaReceptor	209	452
TNFalpha	239	473
Wnt	106	220

Table S1: Node sizes and edge sizes of NetPath pathways were listed**.** 32 curated signaling pathways, associated with cancer and immune responses were utilized for assessing performances and optimization of reconstruction methods and interactomes. Wnt, TCR, TGFbetaReceptor, TNFalpha pathways were used in the parameter tuning process. The remaining 28 pathways were employed in the performance metrics for both network propagation and reconstruction.

Databases	Correlation Coefficient	Node Count	p-Value
PathwayCommons	0.621743	13907	0.000000e+00
ConsensusPath	0.556422	12897	0.000000e+00
HIPPIE	0.613702	11924	0.000000e+00
iREF	0.508434	8473	0.000000e+00
STRING	0.250834	6800	4.549525e-98
OmniPath	0.396779	5134	3.534335e-193

Table S2: The correlation results of pathways over publication count are stated here. All interactomes have a positive correlation with the publication counts. The highest correlation and published node coverage belong to PathwayCommons while the lowest coverage and correlation seem in OmniPath.

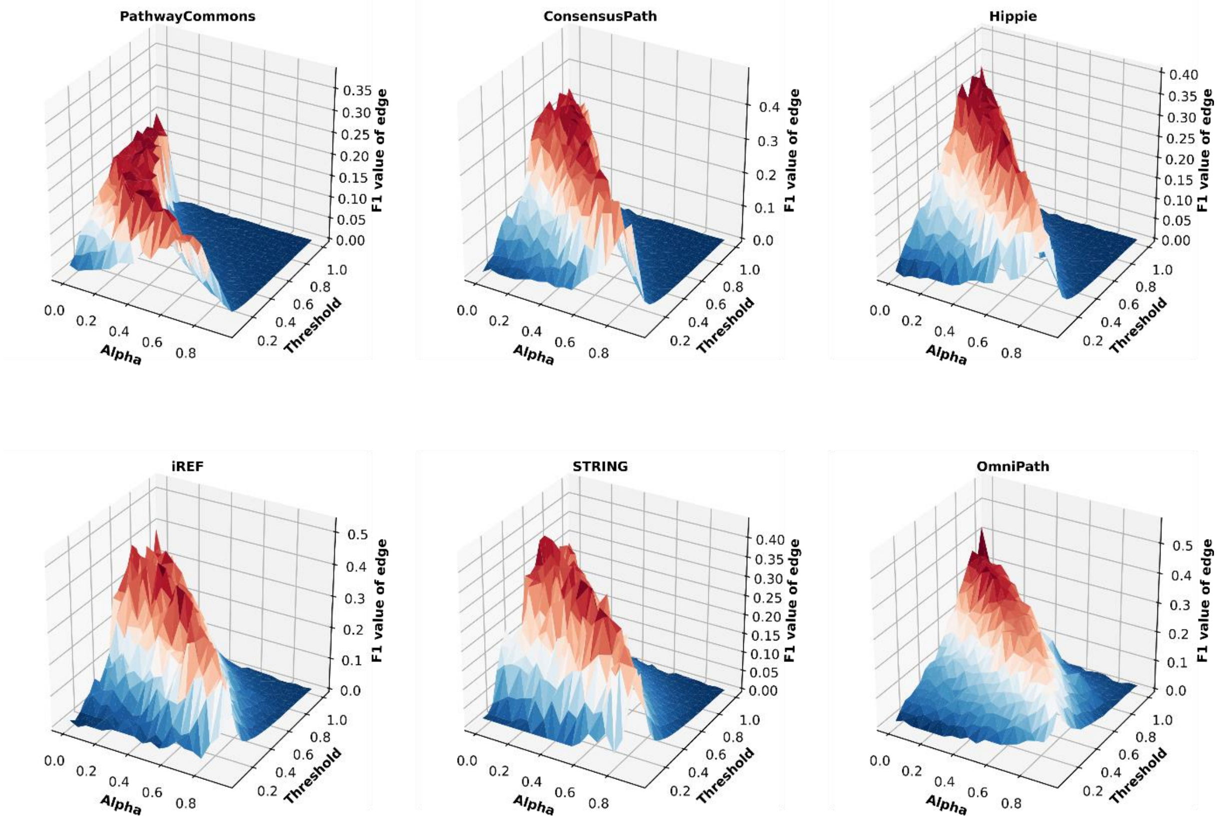

Figure S1: Validation of PageRank Flux (PRF) parameters were performed over F1 scores. The red regions on the graph represent the highest F1 scores of validation parameters while the lowest F1 scores are shown in the blue regions and white regions are transition regions. Damping Factor (**α**) and threshold **(τ)** values, setting off parameters in PRF, were optimized with a grid box where each parameter varied from 0 to 1 with 0.05 increment. Damping Factor can be defined as the probability of visiting the other nodes while the threshold value is defined as the percentage of total flux on the model. During optimization, parameters belonging to the highest 10 F1 scores for each chunk of validation pathways were collected in a hit pool. The mean of both parameters for each interactome was used as optimized parameters.

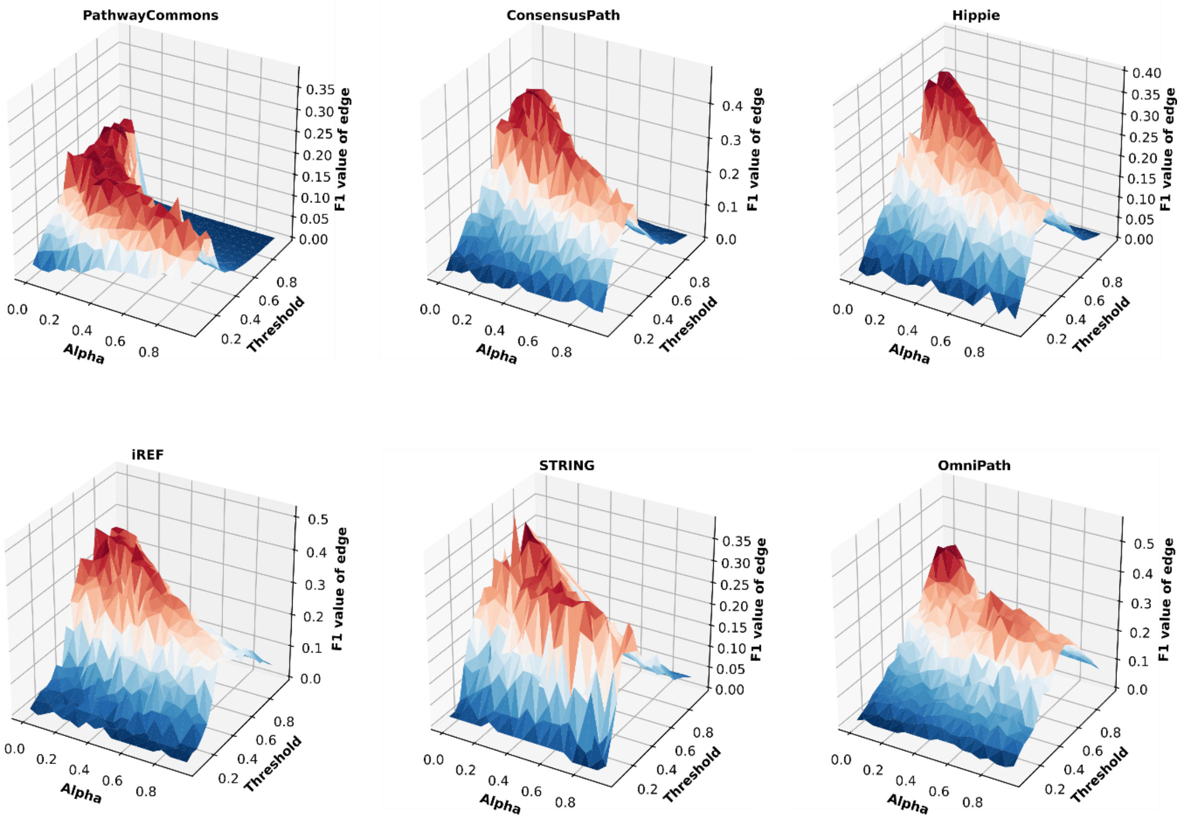

Figure S2: Validation of Heat Diffusion Flux (HDF) parameters were performed over F1 scores. Similarly, Red, blue, and white regions on graphs represent respectively the highest F1 scores, the lowest F1 scores, and transition regions. During optimization, both heat diffusion rate (***α***) and threshold **(τ)** values were varied from 0 to 1 with 0.05 increment. The highest 10 F1 scores for each chunk of validation pathways were considered in the optimization. The means of parameters for each interactome were employed in pathway reconstructions.

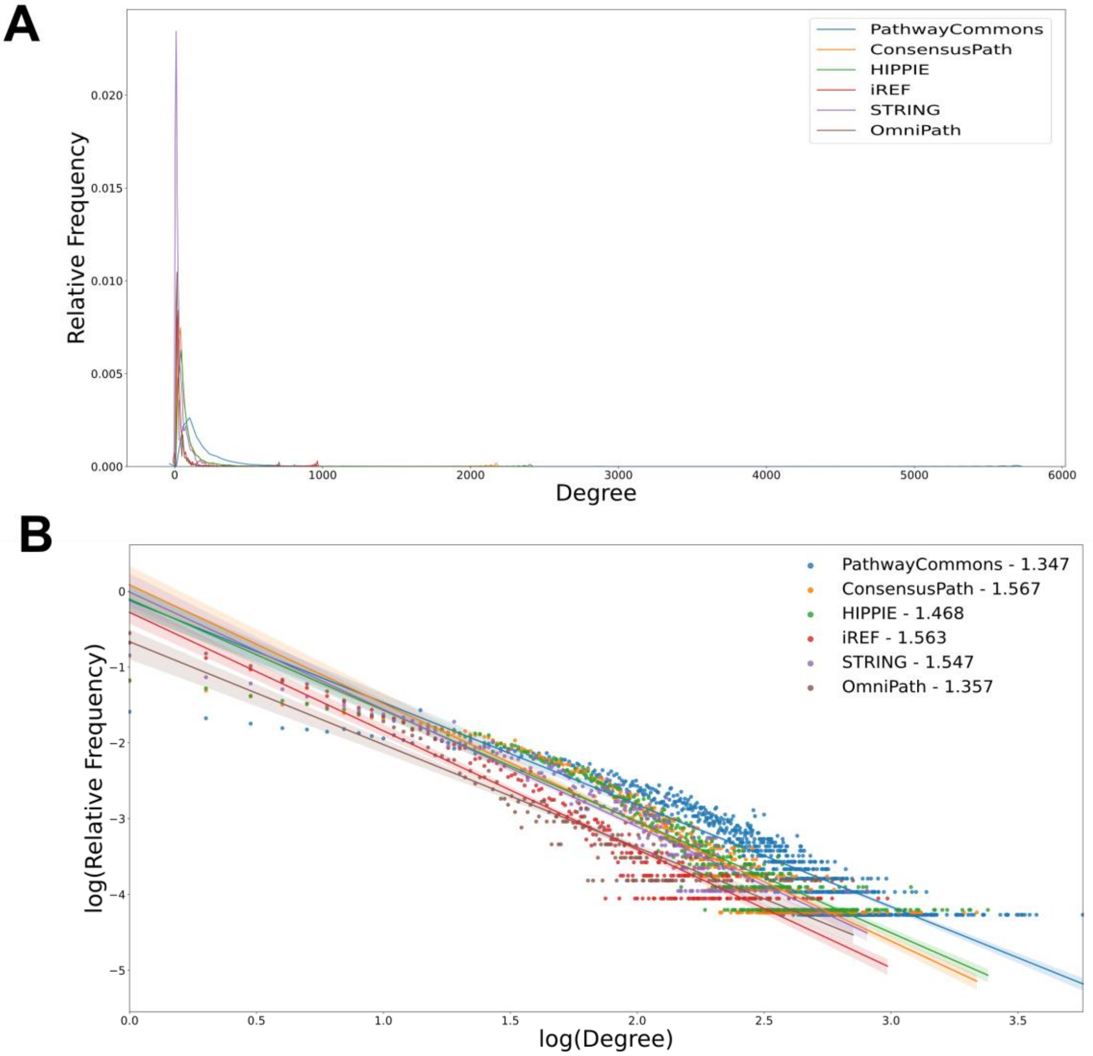

Figure S3: (A) the representation of degree distribution of degrees over interactomes draws a right-skewed graph, which demonstrates most of the nodes are weakly connected interactomes. The resolution of the graph seems low since biological systems follow a power law. **(B**) Log-log scale representation of degree frequencies of interactomes is demonstrated. Indeed, biological networks are scale-free networks. The frequency of degree (P(k)) changes with negative power(-α) of degrees(k). Logarithms of frequency and degree linearized the relationship between degree and its own frequency. Inverse proportions are recognized between degree and its frequency in graphs. Clearly, it seems that PathwayCommons among interactomes have a relatively high frequency of highly connected nodes,

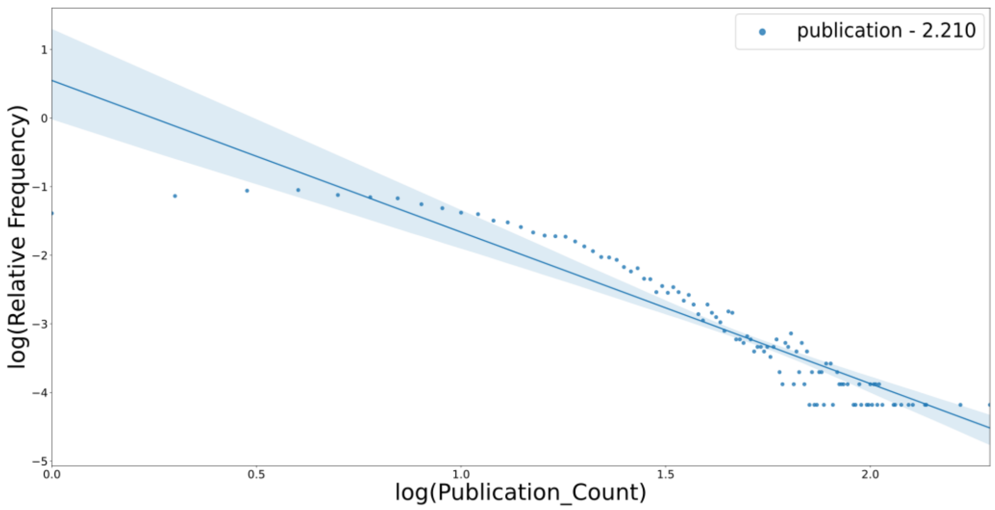

Figure S4: log-log graph of publication distribution shows that the publication of proteins in NCBI follows a power law, similar to degree distribution. Some critical proteins get high attention and are studied more than other proteins. In fact, the highly studied proteins can cause bias on interactome via incorrect edge insertion.

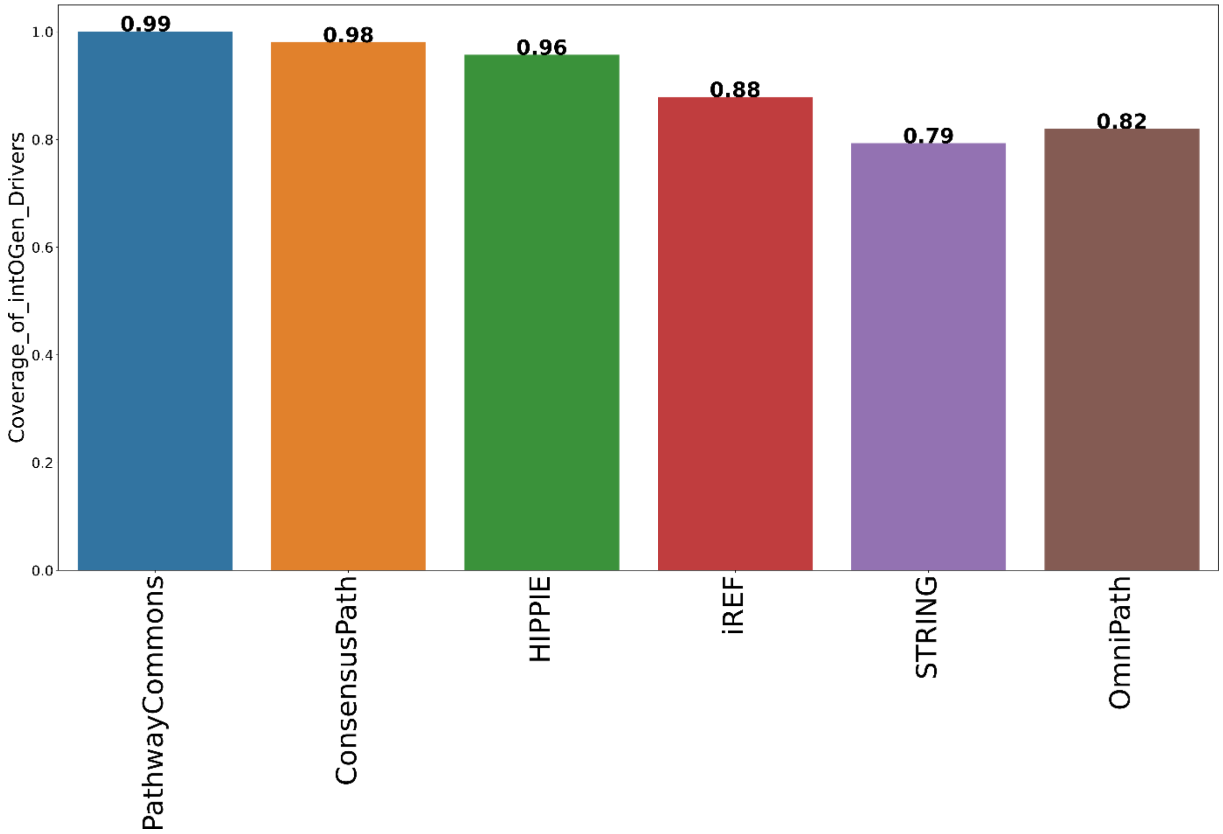

Figure S5: Coverage of Cancer Driver Genes (CDGs) in intOGen Database over interactomes are represented in the boxplot. PathwayCommons, ConsensusPath and HIPPIE efficiently covers CDGs while other interactomes also have a high coverage ratio.

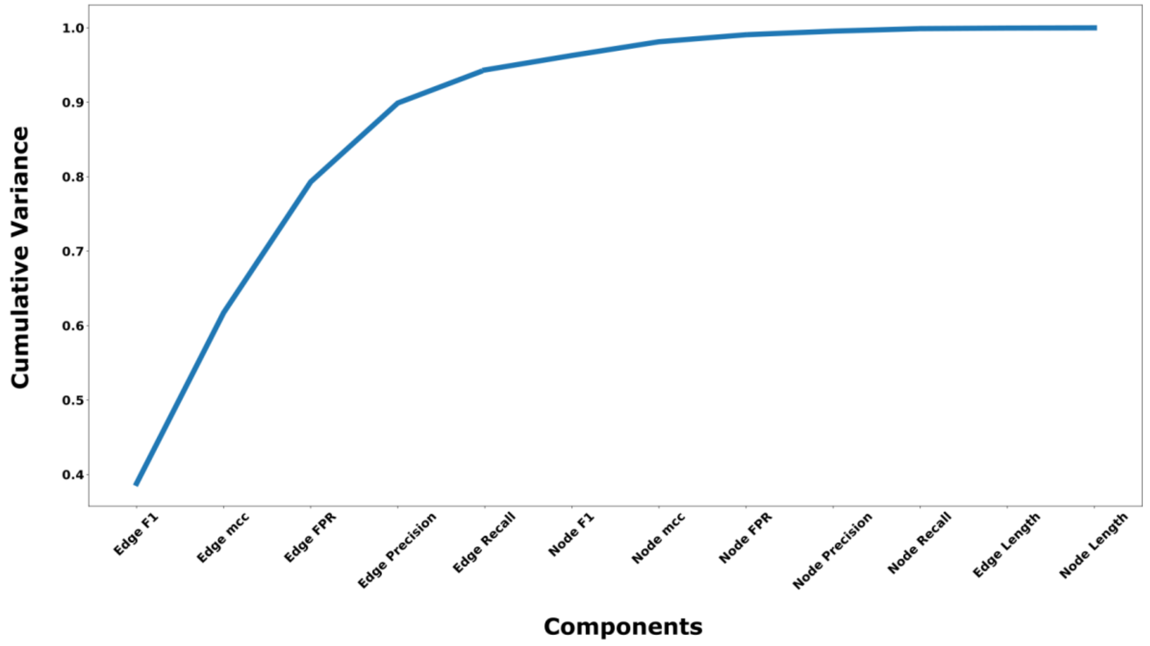

Figure S6: Principal Component Analysis (PCA) over edge-based and node-based scores reveals that more than 90% of the variance is explained by edge-based scores

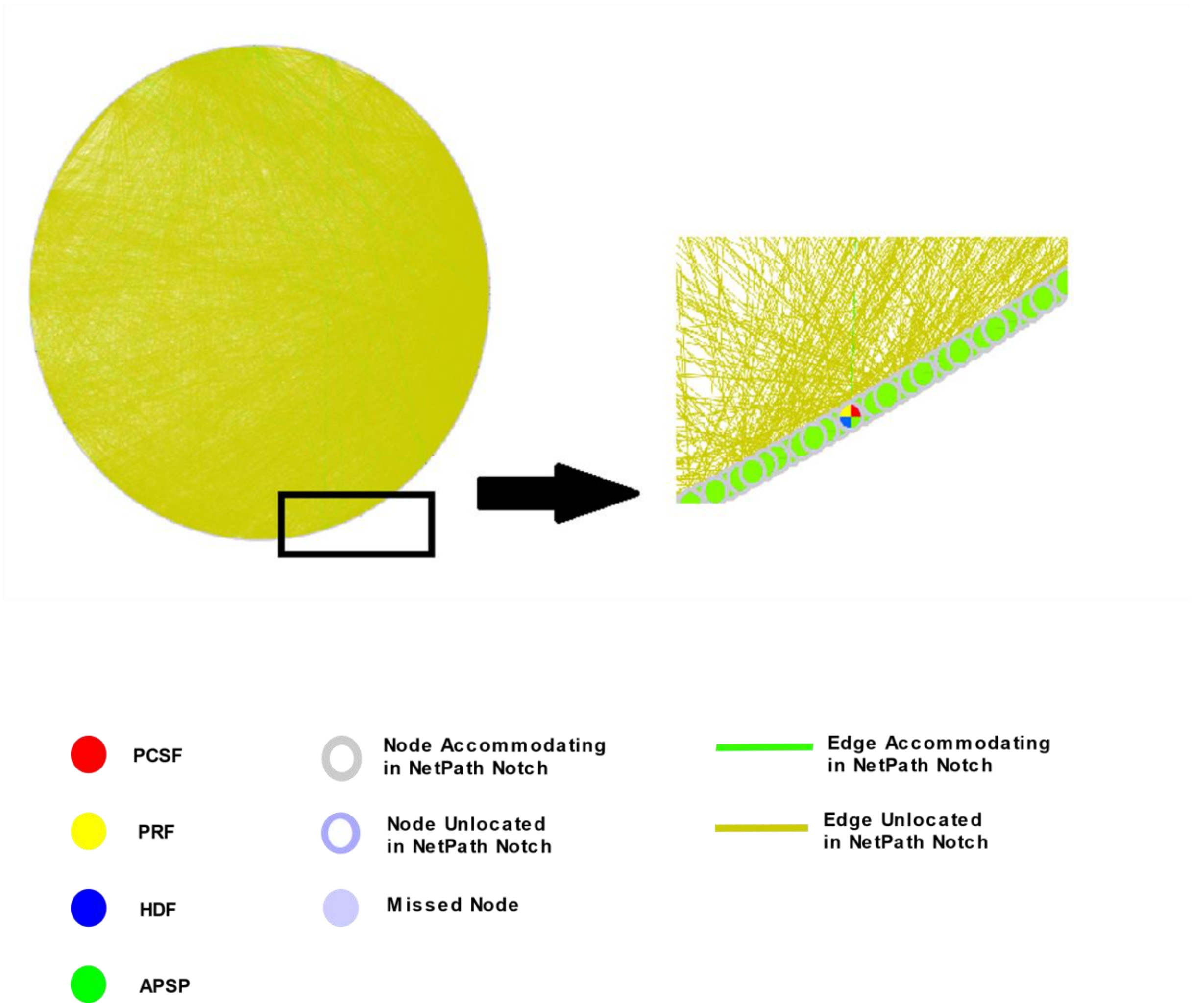

Figure S7: The reconstructed notch pathways are joined with all generated nodes and edges. Reconstruction methods can be seen in nodes that are in red, yellow, blue, and green, respectively showing PCSF, PRF, HDF, and APSP. Edges accommodated in the reference pathway are represented with green linkage while yellowish color represents false constructed edges. APSP reconstruction methods generate a vast number of incorrect edges and nodes.
